# *PIK3CA* mutation and colorectal cancer precision medicine

**DOI:** 10.18632/oncotarget.15724

**Published:** 2017-02-25

**Authors:** Tsuyoshi Hamada, Jonathan A. Nowak, Shuji Ogino

**Affiliations:** Department of Oncologic Pathology, Dana- Farber Cancer Institute and Harvard Medical School, Division of MPE Molecular Pathological Epidemiology, Department of Pathology, Brigham and Women's Hospital and Harvard Medical School and Department of Epidemiology, Harvard T.H. Chan School of Public Health, Boston, MA, USA

**Keywords:** clinical outcome, colorectal neoplasms, molecular pathological epidemiology, non-steroidal anti-inflammatory drugs, tumor microenvironment

The pathogenesis of colorectal adenocarcinoma is driven by the accumulation of multiple genetic and epigenetic aberrations, and is influenced by various endogenous and exogenous factors [1] [Kudryavtseva AV, et al. Oncotarget. 2016;7:53959-53983]. Phosphatidylinositol-4,5-bisphosphonate 3-kinase (PI3K) serves as a major signaling hub downstream of epidermal growth factor receptor (EGFR). The PI3K signaling pathway plays a key role in the pathogenesis of many cancer types, including colorectal cancer. Gain-of-function mutations in *PIK3CA* (the phosphatidylinositol-4,5- bisphosphate 3-kinase catalytic subunit alpha gene) up-regulate the downstream AKT-MTOR signaling pathway, thereby promoting cancer cell growth and proliferation. Mutations in *PIK3CA* exons 9 and/or 20 are present in 10- 20% of colorectal cancers and are associated with other molecular alterations, including *KRAS* mutations and high-degree CpG island methylator phenotype [2] [Rosty C, et al. PLoS One. 2013;8:e65479]. Interestingly, the proportion of *PIK3CA*-mutant colorectal cancer appears to increase gradually from the rectum to the cecum rather than showing an abrupt change at the splenic flexure, supporting the “colorectal continuum theory” [3] and the role of the gut microbiota in carcinogenesis [4] [Ericsson AC, et al. Oncotarget. 2015:6:33689-33704; Wei Z, et al. Oncotarget. 2016:7:46158-46172]. Understanding the pathogenic heterogeneity between *PIK3CA*-mutant and *PIK3CA*-wild-type tumors may explain differences in treatment response between individual colorectal cancers.

In this issue of *Oncotarget*, Ziv and colleagues report a retrospective study of yttrium-90 radioembolization for colorectal cancer liver metastases that suggested differential treatment response according to PI3K pathway gene mutation status (i.e., *PIK3CA*, *AKT1*) [5]. Among 40 patients with metastatic disease, *PIK3CA* and *AKT1* mutations were observed in nine (23%) and one (2.5%) patients, respectively. Patients with mutations in either *PIK3CA* or *AKT1* had a lower cumulative incidence of local progression after radioembolization compared with wild-type patients (55% vs. 92% at 1-year post-embolization). This result could not be solely attributed to differences in tumor aggressiveness by *PIK3CA* mutation status [2]; therefore, the findings suggested that mutational activation of the PI3K pathway may enhance radio-sensitivity of colorectal tumors. Although small, this study has potential therapeutic implications. First, only a fraction of patients incur survival benefit from radioembolization, yet they are all subjected to an invasive procedure. Therefore, it would be clinically beneficial to identify the target population that might respond best to radioembolization. PI3K pathway mutation status may serve as such a biomarker to predict benefit from radioembolization. Furthermore, tumors harboring mutations in the PI3K pathway may be less susceptible to treatment using anti-EGFR antibodies, including cetuximab and panitumumab, which is the current mainstay for targeted therapy of metastatic colorectal cancer [Zhao B, et al. Oncotarget. 2016]. This suggests that PI3K pathway mutation status should be considered in the choice of anti-EGFR therapy, as is currently done for *KRAS* mutation status. The findings of the current study suggest that radioembolization may be an effective treatment modality for this specific subgroup that is already more likely to be resistant to anti-EGFR therapy.

The PI3K pathway interacts with other key signaling pathways in colorectal cancer. Therefore, survival benefits associated with inhibition of other pathways may differ by *PIK3CA* mutation status. Aspirin, a nonsteroidal anti-inflammatory drug (NSAID), inhibits PTGS1 (cyclooxygenase-1) and PTGS2 (cyclooxygenase-2), and aspirin use has shown to reduce colorectal cancer incidence and mortality [Drew DA, et al. Nat Rev Cancer. 2016;16:173-186]. Building on this observation, our previous population-based study reported that postdiagnosis aspirin use is associated with long patient survival specifically in *PIK3CA*-mutant cancer [6]. In this context, aspirin may suppress colorectal cancer progression through inhibition of PTGS2 and prostaglandin E2 synthesis, which are enhanced by activated PI3K signaling. More generally, our findings support the notion that colorectal cancer is a molecularly heterogeneous disease and that molecular classification should be routinely employed to match tumors with the therapy to which they are most likely to respond.

Molecular pathological epidemiology (MPE) can be a powerful tool to decipher differences in treatment outcomes according to patterns of molecular alterations [7] [Rescigno T, et al. Molecules. 2017]. Conventional epidemiology investigates the association of an exposure of interest with a given single disease (here, colorectal cancer). In contrast, MPE utilizes molecular pathology diagnostics for disease subgrouping and examines the association of an exposure with each specific subtype (here, *PIK3CA*-mutant or *PIK3CA*-wild-type colorectal cancer) [1]. Through this paradigm change, MPE research can 1) refine the effect estimate of the association of an exposure with a specific subtype; 2) provide evidence on underlying causality; and 3) help develop personalized strategies of prevention and treatment. Owing to its flexible and integrative nature, MPE can be readily integrated with other traditionally distinct disciplines. For instance, the abovementioned aspirin study illustrates the successful integration of MPE with pharmacoepidemiology (“pharmaco-MPE”) [8]. More recently, integrations with immunology and microbiology have given rise to the subfields of “immuno-MPE” and “microbial MPE”, respectively [8]. Of note, the MPE methodology can be applied to a wide spectrum of human cohort studies, irrespective of study designs (hospital-based or population-based) and disease categories (non-neoplastic diseases as well as neoplastic diseases).

**Figure 1:**
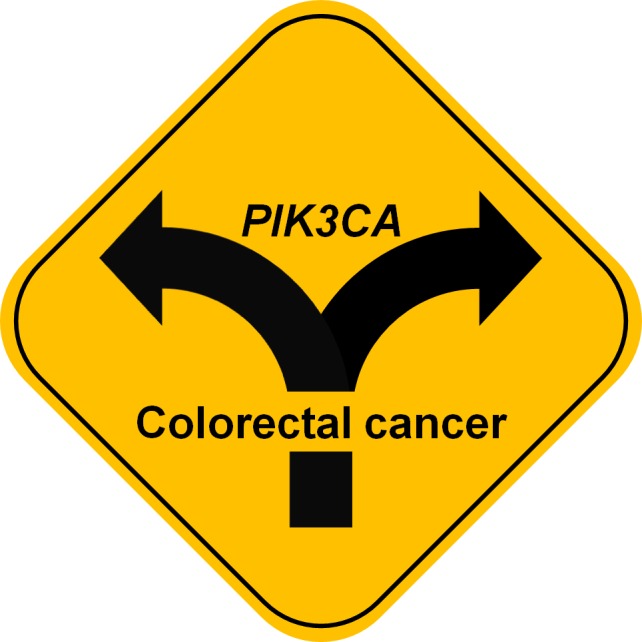
Tumor *PIK3CA* mutation status as a biomarker to predict treatment response of colorectal cancer

In summary, the current study highlights the importance of considering the inter-tumor heterogeneity in clinical studies on colorectal cancer treatment response. This study identifies a potentially novel use for such testing, namely, stratification according to estimated benefit from radioembolization. Since *PIK3CA* is one of the driver oncogenes in colorectal carcinogenesis, we believe that MPE research focusing on the PI3K pathway has the potential to identify predictive biomarkers for response to other treatments (Figure 1), and to guide the development of targeted therapies for precision medicine.
